# Interaction between Stem Cells and the Microenvironment for Musculoskeletal Repair

**DOI:** 10.1155/2020/7587428

**Published:** 2020-03-20

**Authors:** Yong-Can Huang, Zhen Li, Jun Li, Feng-Juan Lyu

**Affiliations:** ^1^Shenzhen Engineering Laboratory of Orthopaedic Regenerative Technologies, Department of Spine Surgery, Peking University Shenzhen Hospital, Shenzhen 518036, China; ^2^National & Local Joint Engineering Research Center of Orthopaedic Biomaterials, Peking University Shenzhen Hospital, Shenzhen 518036, China; ^3^AO Research Institute Davos, Davos, Switzerland; ^4^Department of Orthopedic Surgery, Rush University Medical Center, Chicago, IL 60612, USA; ^5^Guangdong General Hospital (Guangdong Academy of Medical Sciences), South China University of Technology-the University of Western Australia Joint Center for Regenerative Medicine Research, School of Medicine, South China University of Technology, Guangzhou, China

Manipulation of the tissue microenvironment has become a promising method to enhance the regenerative abilities of stem cells/progenitors for musculoskeletal repair. The composition of the microenvironment is determined by the resident cells, extracellular matrix, cytokines, and chemokines as well as the biomechanical property and nutrient status. The microenvironment is altered by the homeostasis and degenerative stage of the native tissue. All the alterations of the surrounding host microenvironment will definitely change the biology of the implanted stem cells/progenitors. Thus, a thorough understanding of the stem cell-microenvironment communication would therefore accelerate the success of musculoskeletal repair. In this special issue with the theme “Interaction between stem cells and the microenvironment for musculoskeletal repair,” we are very pleased to present the 11 accepted papers (7 research articles and 4 review articles) in which how the microenvironment affects the stem cells/progenitors was well investigated.

Bone marrow mesenchymal stem cells (BM-MSCs) play a central role during the process of bone modeling and remodeling; yet, their biology is regulated by the cytokines, biomechanical stimuli, and other types of cells. Increased B lymphopoiesis was found in mouse with osteoporosis [1], which constitutes a microenvironmental factor for BM-MSCs. The study of Pan et al. checked the involvement of activated B lymphocytes in osteoporosis by first establishing two rat models of osteoporosis by ovariectomy or splenectomized variectomy. Examination at 3 months postsurgery showed that both models exhibited signs of osteoporosis, represented by loss of bone volume and quality, as well as the activation of B lymphocytes, represented as increased proportion of CD3-CD45RA dual positivity in bone marrow cells, while SPX alone induced activation of B lymphocytes but did not induce osteoporosis. Further cell study revealed that lipopolysaccharide pretreated B lymphocytes suppressed the mineral deposition and alkaline phosphatase activities of BM-MSCs under osteogenic inductive condition, suggesting impaired osteogenesis, while untreated normal B lymphocytes had no such effect. On the other hand, the suppression of osteogenesis of BM-MSCs could be relieved by the addition of a dexamethasone or Notch inhibitor. These results suggest that bone loss after menopause may have resulted from activated B lymphocytes which have a negative impact on the osteogenesis of BM-MSCs and that the dexamethasone or Notch inhibitor could reduce bone loss through suppressing B lymphocytes. Glutamine provides the energy demand for the cells and serves as a secondary metabolic regulator in bone homeostasis. Dr. Zhou and coauthors contributed a review paper in this issue to majorly summarize the recent evidences regarding how glutamine metabolism mediates the bioenergy of BM-MSCs and how glutamine influences proliferation, differentiation, and mineralization. The authors suggested that extensively basic and clinical investigations are needed to deeply understand the importance of glutamine metabolism in bone homeostasis and to develop new therapeutic strategies. To facilitate the osteogenesis of rat BM-MSCs *in vitro*, Dr. Ni's group used 20 IU/ml erythropoietin combined with the cyclic mechanical stretch (1 Hz sinusoidal curve set at 10% elongation); the underlying mechanism may be associated with the activation of ERK1/2 signaling pathway. Dr. Huang's group found that 10 mM strontium (a trace element in the bone tissue) was able to promote the osteogenic gene expression and mineralization of human BM-MSCs and placenta-derived MSCs. Interestingly, using the exosomes derived from the osteogenically differentiated adipose-derived stem cells (ADSCs), obvious enhancements in the osteogenic differentiation of ADSCs were recorded. Dr. Yang and colleagues further determined that this enhancement was related to the upregulated (201) and downregulated (33) exosomal miRNAs derived from the osteogenically differentiated ADSCs. Hence, beyond the resident cells, the bioactive compounds, and the biomechanical stimuli, exosomal miRNAs probably became a new target to enhance the osteogenesis of endogenous and exogenous stem cells/progenitors.

During the cause of intervertebral disc (IVD) degeneration, gradual changes in the disc morphology, matrix composition, and microenvironment have been observed [2, 3]. In the review paper contributed by Dr. Vadalà and coauthors, the updated knowledge regarding the microenvironment in the healthy and the degenerated IVD was described and how the components of IVD microenvironment regulated the MSC viability and biological potential was summarized. The authors further emphasized the consideration of IVD microenvironment before developing MSC-based and biomaterial-based therapies. The IVD microenvironment is also affected by the phenotype of native resident cells, which can change during aging. Cheng et al. isolated nucleus pulposus (NP) cells from the caudal IVD of young (2 months) and old (24 months) SD rats; the NP cells from old rats showed a senescent phenotype, as well as declined cell proliferation and migration capacity. The transcriptomes of the young and senescent NP cells were analyzed by microarray. A total of 1038 differentially expressed genes were reported between the young and senescent NP cells, with the upregulated genes mainly enriched in the TNF signaling pathway and downregulated genes enriched in the extracellular matrix receptor interaction. The results revealed the underlying genes and pathways of senescent NP cells that were supposed to increase with aging. The reported findings provide a rich transcriptomic dataset for young *vs.* senescent NP cells, which may assist the development of novel biological methods to treat degenerative disc diseases.

The tendon is to transmit muscular forces to the bone, permitting joint motion and subsequent body movement [4]. It was thought that the tendon only consists of tenocytes; nevertheless, the recent studies demonstrated that human and mouse tendons contain stem cells, namely, tendon stem/progenitor cells (TSPCs) [5]. In the review contributed by Dr. Zhang and colleagues, the recent advances in the identification and characterization of TSPCs and their interactions with extracellular matrix and mechanical loading were summarized; meanwhile, the authors outlined the challenges in understanding TSPC biology and function *in vivo* due to the heterogeneity and lack of specific markers and suggested that the mechanobiology of TSPCs and its role in tendon development, growth, repair, and pathology need to be better clarified.

The interaction between stem cells and local microenvironment goes in both directions. Not only the microenvironment can impose on the fate of stem cells, but also stem cells can positively affect the local microenvironment of injured tissues. In Fang's study using a rat peripheral nerve injury model, except from generating neurons, transplanted embryonic spinal cord cells were found to have a regulatory effect on local Schwann cells in the distal nerve and induced them to produce proximal axons to facilitate nerve regeneration. Amyotrophic lateral sclerosis (ALS) is a progressive disease that affects nerve cells in the brain and spinal cord, causing loss of muscle control. Due to the low bioavailability and short half-life *in vivo*, in clinics, the expected outcomes of the intrathecal administration of neurotrophic factors alone was hard to achieve. Pawlukowska et al. performed a clinical study using the autologous lineage negative (Lin^−^) stem cells to treat ALS. The authors thought Lin^−^ stem cell-based therapy would be a reasonable and promising alternative for classic ALS treatments because the Lin^−^ stem cells could produce the trophic support for the host's neurons, stimulate the secretion of neurotrophins (NTs), and differentiate into oligodendrocyte progenitor cells or neurons. In this article, the authors completed a clinical trial to assess the impact of intrathecal administration of bone marrow Lin^−^ stem cells in 32 patients suffering from ALS on articulation; it was demonstrated that 6 patients achieved the improvement of articulation after 28 days, 23 patients remained stable, and 3 deteriorated. Although some valuable findings were observed, several limitations should be acknowledged such as the small number of patients, the lack of control group, and a short observation period.

During muscle regeneration, as reviewed by Dort et al., the spatial recruitment of proinflammatory and anti-inflammatory macrophages is different in addition to their temporal recruitment; proinflammatory macrophages are located close to proliferating satellite cells, while anti-inflammatory macrophages are found close to the regenerating area containing differentiated myoblasts. Depletion of proinflammatory macrophages resulted in impaired muscle regeneration in animal models. The suppression of the switch of macrophage from proinflammatory phenotype to anti-inflammatory phenotype reduced muscle fiber growth but did not affect the clearance of necrotic tissues. At the cellular level, proinflammatory macrophages promote myoblast proliferation and inhibit differentiation, while anti-inflammatory macrophages inhibit myoblast proliferation and stimulate their differentiation and myofiber growth. Direct coculture of macrophages also promoted proliferation and inhibited apoptosis of myogenic cells. Altogether, these findings suggest that different subsets of macrophages have complementary roles in the regulation of satellite cell/myoblast function, myogenesis progression, and optimal muscle regeneration.

In summary, the published articles in this special issue add new perspectives to the understanding regarding the interaction between stem cells/progenitors and the microenvironment for the bone, intervertebral disc, tendon, muscle, and nerve regeneration. We hope that all these basic and clinical studies will be helpful for the development of stem cell-based therapies for musculoskeletal repair. Finally, we would like to express our gratitude to all the authors, the reviewers, and the editorial board members of this journal for their contribution and assistance to make this special issue successful.
